# Biocatalysis in the Recycling Landscape for Synthetic Polymers and Plastics towards Circular Textiles

**DOI:** 10.1002/cssc.202002666

**Published:** 2021-02-12

**Authors:** Christina Jönsson, Ren Wei, Antonino Biundo, Johan Landberg, Lisa Schwarz Bour, Fabio Pezzotti, Andreea Toca, Les M. Jacques, Uwe T. Bornscheuer, Per‐Olof Syrén

**Affiliations:** ^1^ RISE Research Institutes of Sweden Argongatan 30, Box 104 SE-431 22 Mölndal Sweden; ^2^ Department of Biotechnology and Enzyme Catalysis Institute of Biochemistry University of Greifswald Felix-Hausdorff-Strasse 4 17487 Greifswald Germany; ^3^ School of Engineering Sciences in Chemistry Biotechnology and Health KTH Royal Institute of Technology Science for Life Laboratory Tomtebodavägen 23, Box 1031 171 21 Solna Stockholm Sweden; ^4^ School of Engineering Sciences in Chemistry Biotechnology and Health Department of Fibre and Polymer Technology KTH Royal Institute of Technology Teknikringen 56–58 100 44 Stockholm Sweden; ^5^ Swedish Stockings Tyskbagargatan 7 114 43 Stockholm Sweden; ^6^ The LYCRA Company UK Limited 60, Clooney Road, Maydown Londonderry N. BT47 6TH Ireland; ^7^ KTH Royal Institute of Technology School of Engineering Sciences in Chemistry, Biotechnology and Health Wallenberg Wood Science Center Teknikringen 56–58 100 44 Stockholm Sweden; ^8^ Present address: REWOW srl Via Cardinale Agostino Ciasca 9 701 24 Bari Italy; ^9^ Present address: Hyper Island Virkesvägen 2 120 30 Stockholm Sweden

**Keywords:** biocatalysis, enzyme engineering, plastics, recycling, textile

## Abstract

Although recovery of fibers from used textiles with retained material quality is desired, separation of individual components from polymer blends used in today's complex textile materials is currently not available at viable scale. Biotechnology could provide a solution to this pressing problem by enabling selective depolymerization of recyclable fibers of natural and synthetic origin, to isolate constituents or even recover monomers. We compiled experimental data for biocatalytic polymer degradation with a focus on synthetic polymers with hydrolysable links and calculated conversion rates to explore this path The analysis emphasizes that we urgently need major research efforts: beyond cellulose‐based fibers, biotechnological‐assisted depolymerization of plastics so far only works for polyethylene terephthalate, with degradation of a few other relevant synthetic polymer chains being reported. In contrast, by analyzing market data and emerging trends for synthetic fibers in the textile industry, in combination with numbers from used garment collection and sorting plants, it was shown that the use of difficult‐to‐recycle blended materials is rapidly growing. If the lack of recycling technology and production trend for fiber blends remains, a volume of more than 3400 Mt of waste will have been accumulated by 2030. This work highlights the urgent need to transform the textile industry from a biocatalytic perspective.

## Graphical abstract

**Circular textiles**: This Review highlights the urgent need to transform the textile industry from a biocatalytic perspective to enable fiber to fiber recycling of today's complex textile materials (renewable building blocks schematically represented by molecules sketched). If the production trend for synthetic textile fiber blends remains, a volume of more than 3400 Mt of waste will have been accumulated by 2030.

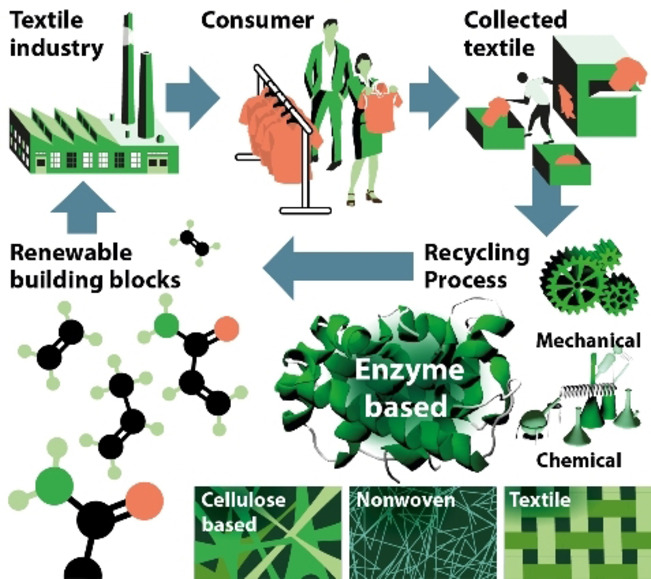

## 1 Introduction

The plastic problem constitutes one of the most pressing challenges that our society faces today, which is perhaps well illustrated by the fact that it has been projected that there will be more plastic particles than fish by weight in our oceans by 2050.^[1]^ Up to now, around 8.3 billion tons of synthetic polymers and plastics have been manufactured,^[2]^ out of which only 9 % have been recycled, mainly due to associated unresolved technical challenges^[3]^ and the comparative costs between recycled and virgin materials. Textiles correspond to one of the largest application areas for synthetic polymers^[2]^ and the extensive and increasing use of plastics in garments is of growing environmental concern. In 2017 the textile global market size surpassed 103 million tons (Mt), out of which 63 % constituted petroleum‐based virgin fibers.^[4]^ Reaching a circular textile economy^[5]^ requires more efficient use of resources and increased input of bio‐based feedstock,^[6]^ but also radically improved recycling technologies.^[3a]^ In particular, separation of individual components from complex polymer blends, to enable regeneration of fibers with the same properties as the respective starting materials, remains a hurdle.^[7]^ Fiber‐to‐fiber recycling constitutes a major unresolved challenge in the field, which severely restricts recycling possibilities of used textiles to mainly downcycling applications, where possible.^[3b]^ Hence, only a low percentage of all used textiles world‐wide are recycled and around 73 %^[5]^ are being incinerated or disposed into landfills after use. Due to the low recycling rates, approximately 350 Mt of petroleum is needed each year to sustain the polymer industry with virgin building blocks; a number expected to double within the next few decades.^[2]^

The large and increasing[^1a^, ^2^] demand for fossil‐based resources for the production of synthetic polymers and fibers is associated with resource depletion, pollution, and release of CO_2_ that contributes to global warming and environmental consequences associated with post‐consumer waste.^[8]^ At present, only 1 % of all synthetic polymers are generated from renewable sources,^[9]^ a fact that does not align with the now established United Nation's sustainable development goals (SDG) of the Agenda 2030. Several of these aims relate to the supply chain of the textile industry, such as the goal to establish responsible consumption and production, from raw material extraction to end of life. Considerations of environmental issues related to textile production are well established in legislative frameworks, such as the EU's industrial emissions directive (IED), which provides a reference document.^[10]^ Still, the textile manufacturing industry is characterized by significant emissions of greenhouse gases corresponding to 1.2 billion tons y^−1^,^[5]^ and high resource intensity regarding natural resources and labor, whereas investment costs are comparably low.^[1]^ As a consequence, upstream suppliers are mostly found in regions with low wages and lack of stringent environmental legislation and enforcement. Increasing legislation will help drive circularity, and the EU has already mandated that member states have to collect disposed textiles separately from general municipal waste by 2025. If this action is carried out in a timely manner, then there is a clear scenario that textile waste will be collected at higher volumes than what can be handled by currently installed recycling capacities or available recycling technologies.[^5^, ^11^] Herein, we show that the use of blended materials is rapidly growing, further complicating any textile recycling initiatives as blends obstruct recycling and recovery of raw materials at the end of use. Biotechnology could provide a solution to this pressing problem by selective depolymerization to remove minority fibers acting as contaminants preventing reprocessing, to isolate individual components or even recover monomers. By collecting and analyzing literature and market data in a Review‐like manner in concert with experiments and generation of novel data sets, our work stresses the challenges ahead towards achieving a circular textile economy. Whereas previous Reviews on biocatalytic recycling of plastics^[12]^ mostly focused on packaging applications and bottles, this work highlights the urgent need to transform the textile industry from a biocatalytic perspective.

## 2 Trends in Production of Textile Fibers Show that Blends Containing Synthetic Polymers are Increasing

**Figure 1:**
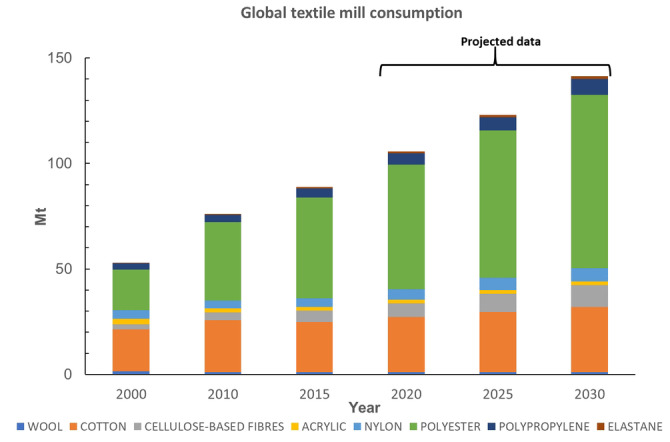
Current and predicted total fiber demand shows a growing market share for synthetic fibers. Data is taken from ref. [13] and from industrial partners (see Experimental Section).

**Scheme 1:**
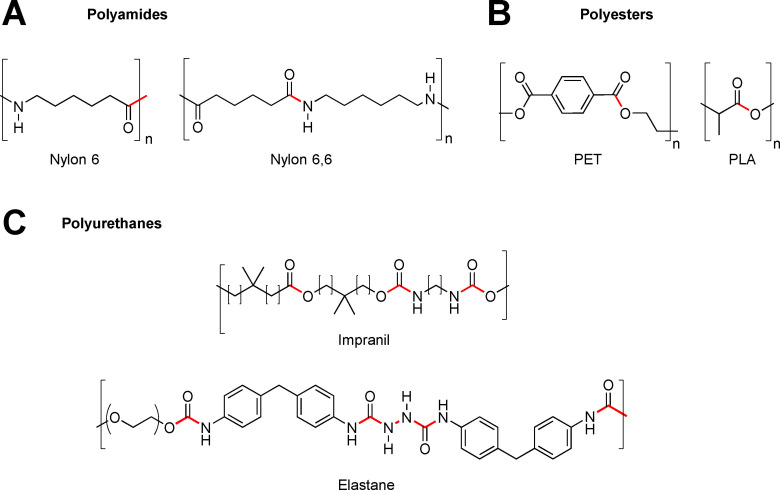
Structures of industrially important synthetic polymers used in textiles (chemical bonds hydrolysable by enzymes are shown in red). The bio‐based polymer PLA is shown for reference.

Textile production is heavily based on petroleum‐derived virgin polymers that currently constitute over half of the textile fiber market (Figure 1, Table S1 in the Supporting Information).^[13]^ The market share for synthetic fibers in textile applications has increased from 55 % in 2000 to around 68 % today (Table S2, Supporting Information). Among synthetic textile fibers, polyethylene terephthalate (PET, Scheme 1), with a yearly production of close to 40 Mt, is one of the most widely used polymers in the global textile industry;^[14]^ according to forecasts its demand in forthcoming years will continue to grow (Figure 1, Table S1).^[2]^ For polyamides (PA), the market share for textile production is around 5 % with a total production today of 5—10 Mt.^[4]^ The increased use of synthetics over natural fibers (wool, cotton, cellulose‐based fibers, Figure 1) illustrates a difficult balance: comfort and durability versus recyclability, at low and affordable cost for textiles that are intended to be used longer than single‐use plastics in packaging applications.

Renewable polyamides and polyesters exist (e. g., from corn starch), including bio‐based PET, polytrimethylene terephthalate (PTT), polylactid acid (PLA), Nylon 6 and Nylon 6,6, and other variants such as Nylon 11. Still, considering textiles, bio‐based polymers have made more penetration into packaging, floorings, and automotive than into apparel.^[15]^ In 2019, bio‐based polyesters represented less than 1 % of the total polyester market despite being introduced over 10 years ago.^[15]^ This lack of penetration is caused by numerous issues including price, fiber and fabric properties (e. g., lower abrasion resistance and melting point for PLA compared to PET), and the difficulty in getting support from the auxiliary business to develop new dyeing and finishing chemicals for emerging/niche fibers.^[15]^

**Figure 2:**
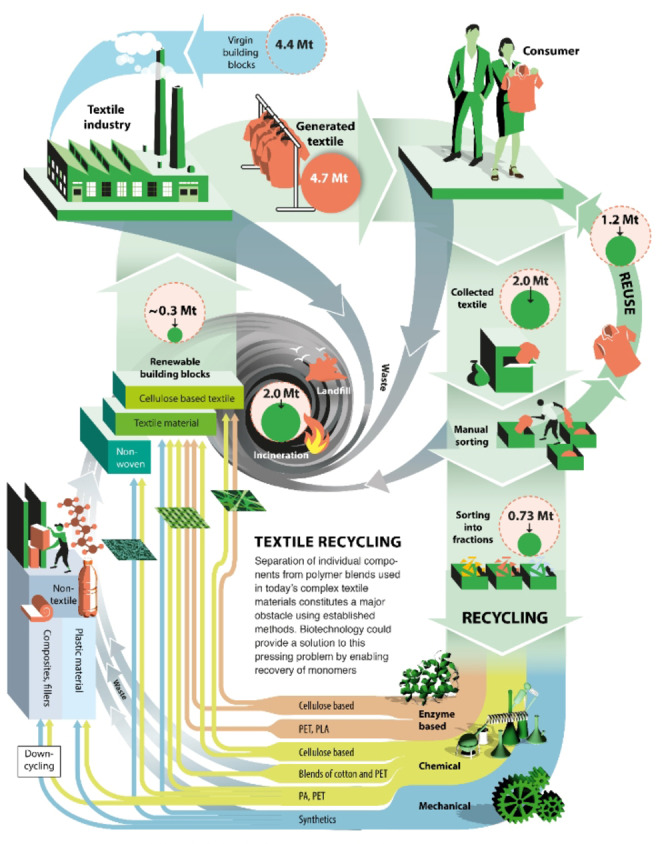
Different recycling routes for collected and sorted textile materials include fiber‐to‐fiber or even textile‐to‐textile recycling. Data is for Northwestern Europe, for which detailed data on composition of textile material flows is available, corresponding to approximately 5–10 % of the global volumes.^[18]^ Chemical recycling includes chemical recycling by depolymerization. With enzyme‐based recycling we refer to processes that incorporate a biocatalyst, chemical pre‐treatment steps could still be key (see below).

Today, the vast array of different fibers available to fashion are decreasingly used as single polymer types, but rather as blends; for instance, as 50 %:50 % cotton (CO) and polyester, 90 %:10 % polyamide and elastane [a poly(urethane‐urea) type polymer, e. g., spandex, Scheme 1, Table S3].^[13]^ This picture has been confirmed in dialogue with Nordic‐based textile brands (Data collection and Survey, Supporting Information). It was evident that the most common fiber blends with elastane content have a polyester, PA, or CO base. Certain applications with wool material may also have some elastane content. For instance, cotton‐based material in denim previously contained an elastane content of 1–2 % but has in some qualities increased to as high as 10 %. Textile blends containing elastane‐ (common in stretch denim, swimwear, sportswear, intimate apparel, and hosiery/leggings)^[16]^ and polyester‐ or nylon‐based fibers are increasing on the market (Figure 1, Tables S1 and S3). Elastane stands out with the largest predicted volume growth of 66 % within the next decade, which can be compared to 39 % for the polyester segment (Table S1). In the 1950’s, DuPont discovered and launched elastane under the brand name LYCRA®. In its early days, elastane was an expensive fiber, with prices as high as $10 kg^−1^ for branded 40 denier elastane in 2012. The price in 2017 has approximately halved to 4.15–4.25 $ kg^−1^ for unbranded fiber, which meant that elastane content within a garment was no longer a cost driven decision. The total market share of elastane is predicted to grow from 0.7 to 1 % by 2030 (Tables S1, S2, S4). Although this quantity seems miniscule, elastane stands out as a specific issue in that, as a minority fiber, it is used in an ever‐increasing number of garments: at present around 20 % of the potentially recyclable textile waste fraction contains elastane,^[17]^ a number that could grow to 29 % by 2030. As blends are increasing, a detailed analysis of textile material flows and composition is important, but still such data sets are scarce. For Northwestern Europe, where data is available, around 40 % of all sorted post‐consumer textile waste that is potentially recyclable is composed of different polymer types.^[18]^ Recycling of some fiber blends is under development, but so far techniques are unproven at scale^[19]^ from both a technology and investment and operations costs perspective. Thus, the major part of all post‐consumer textile waste is either incinerated for energy recovery or disposed of into landfills (Figure 2).^[20]^ From 4.7 Mt of used textiles, the main bulk ends up in household waste and consequently landfills or incineration depending on regional policy. According to studies this may be in the range between 2–3 Mt. Only 2 Mt are collected at the end of life and from that 1.2 Mt holds enough value to be reused. A minor part of the collected textile volume (≈0.75 Mt) can be handled today in our current recycling system, typically by mechanical recycling (Table S5, Supporting Information). In the current situation, up to 5 % of collected textiles represent potential feedstock for textile‐to‐textile recycling (Figure 2). In the vision for a future textile management system, this number will be dramatically increased.

## 3 Recycling Technologies for Blends that Include Synthetic Fibers in Textiles are Lacking

At present, only approximately 1 % of the synthons used for textile production consist of building blocks generated from the textile waste stream.^[5]^ Given the fact of the built‐in value in terms of resources (energy, material, chemicals, water, as well as human capital), today's end‐of‐life‐handling of textiles leads to tremendous loss of valuable material, damage to the environment, and significant emission of greenhouse gases during the material life span.^[21]^

Most secondary raw materials have other sources than textiles, such as PET bottles^[5]^ (for polyester up to ≈10 % of the total production volume^[22]^) or fish nets (for Nylon 6).

One emerging and innovative strategy capitalizes on the high specificity of enzyme catalysis under mild reaction conditions to achieve selective depolymerization of individual components from material blends. The latter has been emphasized for natural fibers (wool^[23]^ and cotton^[24]^), blends containing cotton and polyester (PET),^[19]^ and more recently for textiles with high polyester content.^[25]^ In fact, enzymes have successfully been applied for decades in the textile manufacturing process in order to replace harsh chemicals, such as leather processing (proteases, lipases, amylases), stonewashing (cellulases), desizing (amylases), and bleaching (laccases, catalases). Synthetic biology strategies for textile processing^[26]^ and generation of bio‐based additives, dyes,^[27]^ and sustainable functionalized building blocks from versatile cell factories^[28]^ have recently received significant attention.^[29]^ However, moving from biocatalytic synthesis of small molecules and functionalization of material surfaces to complete depolymerization of the core of synthetic polymers constitute various formidable challenges. Textile materials display a complex microstructure due to, for example, weaving and knitting of components making catalyst accessibility a bottleneck. The polymer microstructures determine its biodegradability with amorphous regions of the polymer that can be degraded, whereas parts of high crystallinity efficiently shield reactive bonds to biocatalyst.^[30]^ For natural fibers, this bottleneck can be overcome by auxiliary enzymes that break strong secondary structure interactions, such as biocatalytic oxidative cleavage of cellulose for enhanced accessibility of cellulases.^[31]^

For polymers containing carbon–carbon main chains (e. g., vinyl‐based materials), significant biocatalytic depolymerization has not been described yet due to the lack of activating functional groups. Furthermore, studies regarding the key mechanistic features as well as the evaluation of the mainly heterogeneous kinetics for enzyme catalysis on even hydrolysable synthetic polymer types are lacking, thereby complicating the challenges to quantify efficacy and optimize the current method for a large scale (industrial) application. Although enzymes suitable for the degradation of pure plastics at an industrial scale have been found, as recently shown for PLA^[32]^ and rapid enzymatic depolymerization of pre‐treated postconsumer PET waste bottles using an engineered esterase,^[33]^ it does not necessarily mean that the same enzymes can degrade mixed fiber textiles at the same efficiency. The polymer properties and the suitable pretreatment methods (Table S5) for textiles might be totally different but determinant for the enzymatic reaction.

At present, mechanical recycling of denim‐, wool‐, and cotton‐based products is possible to some extent.^[19]^ Chemical and mechanical recycling of polyesters to secondary raw material for PET production is in place and can be used to feed‐in lower amounts of sustainable building blocks into the textile value chain.^[22]^ Moreover, pre‐consumer waste within PA and polyester mills with known material flows, quality, and content are currently producing secondary raw material for textile fabrics (≈1 %).^[5]^ These techniques are mainly based on thermal recycling procedures. Post‐consumer polyamide 6 waste from used fish nets can be chemically recycled and by including some separation processes, high quality monomers for re‐polymerization can be retrieved. Chemical and thermomechanical recycling strategies for valorization of plastics and synthetic fibers can suffer from high energy requirements and harsh reaction conditions that can damage the material constituents, which is in particular a disadvantage when separating out a minor constituent such as elastane from another fiber type. Moreover, unspecific cleavage of bonds in the backbones of polymers obstructs downstream separation and purification.

To stress the separation challenge facing the textile value chain, we disclose a study designed to further examine established technologies in regard to how they perform with elastane present in blends. The aim was to achieve fiber‐to‐fiber recycling (Figure 2) using blends of PA and elastane, as blends thereof are commonly found in textiles today (Table S3, Supporting Information). For the study, PA‐based materials (PA6 and PA6.6, also referred to as Nylon 6 and Nylon 6,6 respectively), with a range of different elastane content (5–22 %) were applied in the separation trials. PA6 and PA6.6 (Scheme 1) are the two most common polyamides used in textile and plastic industries. Differences between the materials are, for instance, lower cost and temperature resistance for PA6, while PA6.6 has higher modulus and better wear resistance. The applications of the materials are similar, for example, both as fibers in the textile industries and injection molded parts in the automotive industry. The elastane fibers act as contaminants in the process of producing new fibers. If the contaminants remain in the material when, for example, pulling new fibers during the recycling, this results in low melt strength and breakage. Thus, prior to recycling, removal of the contaminants by separation is important.

Two main separation routes were examined: (i) melt filtration followed by melt spinning and (ii) mechanical separation with consecutive rotor spinning (for detailed description, see the Experimental Section and the Supporting Information, Tables S6 and S7). These separation techniques were chosen since they in theory could handle contaminants, represented here by the elastane content (from 5–22 %). Hypothetically, melt filtration of a melted textile material using an extruder enables the polyamide to melt while the un‐melted elastane should remain in the filter, thus enabling separation (especially for elastane and PA6 which are associated with larger differences in their *T*_m_, values; Experimental Section, Table S8). Melt filtration followed by melt spinning with the current equipment available (see Experimental Section) was, however, not possible since the elastane material was not separated from the polyamide. It was evident that the elastane degraded during the process and therefore passed through the filter. The longer the residence time, the more degradation occurred. With the current equipment and due to the ground fabric being very fluffy, it had to be force‐fed manually into the compounder, resulting in very low feeding speed and thereby longer residence time. The elastane fibers were also very thin; therefore, it was possible for them to go through the available filters. Potentially with a better feeding system (minimizing residence time in the extruder) and a finer filter it could be possible to separate elastane from a material with a different melting temperature. Another possibility is to use the material in other down‐cycling applications, for example, by injection molding, which was further explored. Extruding and melting a material that contains low amounts of elastane was shown to be feasible for injection molding to plastic parts of good quality (Tables S6 and S7, Supporting Information).

The results for the mechanical process revealed several challenges at the scales used in the experiments. For mechanical separation of the polyamide fabric with high elastane content (≈22 %), the material disappeared in the tearing machine. However, during the rotor spinning process using lower elastane content (≈5 %), one could see that elastane was partly separated. Also, some threads were developed during the spinning procedure. Using finer parts of the fabric in the tearing machine followed by a ring spinning technique could possibly ameliorate separation performance. Together, these results emphasize the immense challenges to handle fiber blends containing elastane.

## 4 Biotechnological Recycling Routes for Fiber Blends Require Strong Research Efforts Now

**Figure 3:**
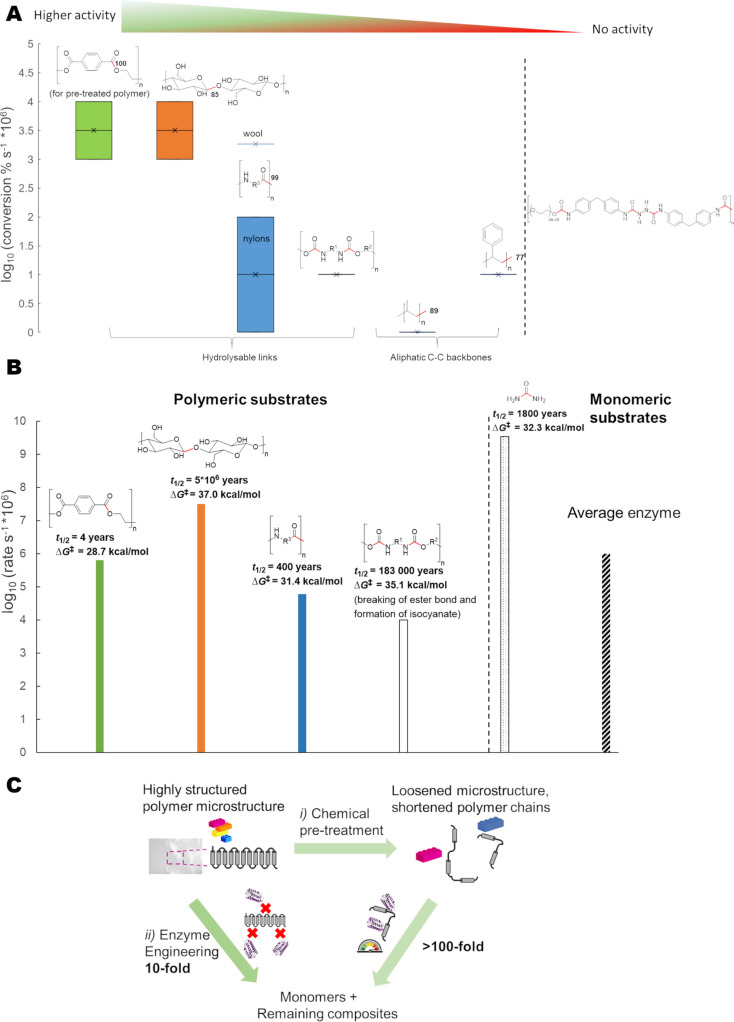
Biocatalytic activity towards plastics used in the textile industry. (A) Compiled experimental conversion data (given as conversion of polymer per time unit, % s^−1^) shown as a logarithmic function for clarity. Inherent reactivity of relevant chemical bonds (shown in red) is given as experimentally determined bond dissociation energies^[53]^ [kcal mol^−1^] of monomeric/oligomeric substrate. For cotton, the bond dissociation energy is represented by a glycosidic ether bond due to lack of experimental data. Experimental data for carbamates was not available as well. Data is shown for (from left to right): pre‐treated PET[^36a^, ^36b^, ^54^] (green bar), cellulose[^31^, ^55^] (i. e., cotton, orange bar), nylon[^41^, ^56^] and wool^[23]^ (blue bar and line, respectively), polyurethane^[57]^ (line, lack of available experimental data prevented calculation of range of conversions). For reference, data for polypropylene[^47^, ^58^] and polystyrene[^50^, ^51^, ^59^] are given. The structure of spandex is shown (right). For details, see Table S8, Supporting Information and the materials and methods. (B) Enzymatic rate constants [s^−1^]. Half‐life and free energy of activation (▵*G* ^≠^) of uncatalyzed hydrolysis reactions are given at 25 °C, for the relevant scissile bond[^34^, ^60^] of the corresponding model dimer/oligomer and compiled from experimental data. The uncatalyzed half‐life of 4 years for PET thus reflects the ester bond in monomeric structure and is significantly higher for polymer. Data is shown for (from left to right): pre‐treated PET[^36a^, ^36b^, ^54^] (green bar), cellulose[^31^, ^55a^, ^55b^, ^55c^] (i. e., cotton, orange bar), nylon[^41^, ^56^] (blue bar), polyurethane^[57]^ (white bar). For reference, data is given for hydrolysis of urea^[61]^ (*k*_cat_, dotted bar) and average enzyme proficiency^[62]^ (in terms of *k*_cat_, striped bar). For details, see Table S8, Supporting Information and the materials and methods. (C) Potential strategies to increase activities shown in (A) and (B) include pre‐treatment of polymer for enhanced accessibility (in analogy to cellulose degradation) and/or enzyme engineering.

Biotechnological methods to re‐generate monomers from synthetic fiber blends are hampered by low enzyme accessibility and lack of existing efficient enzymes capable of expediently decomposing man‐made synthetic materials. We compiled available experimental data for enzyme‐catalyzed depolymerization of polymers used in textiles (Figure 3A,B, Table S3) with an emphasis on materials with hydrolysable bonds. We included data for polyolefins (polypropylene non‐woven fabric) extensively used for manufacturing of personal protective equipment and polystyrene used in construction. Experimental data included in Figure 3 for calculation of polymer conversions (Figure 3A) and enzymatic first‐order rate constants (Figure 3B) were chosen based on the criteria that monomer, or relevant metabolite formation, was demonstrated and analyzed. From Figure 3A we conclude that the conversions calculated by us, expressed as % conversion of polymer to monomer (or metabolites) per second, span four orders of magnitude (note that data for PET‐degradation relates to pre‐treated polymer). Although the reaction condition in each case was different (temperature, composting, fermentation, utilization of whole cells or purified enzymes), we advocate that comparison of the reported optimized yield for each system can give valuable insight and, in fact, points towards the key importance of the material morphology and associated crystallinity in dictating the rate (note that calculations from enzyme initial rates available might not exactly reflect total degradation time). The latter was corroborated by the fact that there is no clear relationship between bond dissociation energies and conversion (Figure 3A). Moreover, calculation of apparent first‐order enzymatic rate constants (when possible from available experimental data) shows that hydrolysis of reacting bonds in relevant polymer substrates was not related to uncatalyzed half‐life of respective dimer/oligomer substrate (or corresponding calculated ▵*G* ^≠^‐values for uncatalyzed hydrolysis, Figure 3B). In fact, the acetal unit found in cellulose is the most inert bond type towards non‐catalyzed hydrolysis with a half‐life of five million years (for β‐methylglucopyranoside model substrates; 22 million years for the hydrolysis of β‐linked glucosepyranoside dimer^[34]^).

Existing microbial enzymes are capable of modifying synthetic polymers^[12a]^ (Figure 3, Table S8), emerging then as potential biocatalysts for recycling of textile fibers. It is reasonable to expect that man‐made polymers with hydrolysable bonds in their backbone structures might be biodegradable by hydrolysis, in analogy to wool and cellulose (Figure 3). Cutinase enzymes (E.C. 3.1.1.74) are well known for their ability to depolymerize cutin, a polyester which forms the cuticle of higher plants. PET is an aromatic polyester of terephthalic acid and ethylene glycol. In 2005, a hydrolase from *T. fusca* was reported to degrade PET to some extent,^[35]^ and since then several reports have been published; in particular, demonstrating PET hydrolysis employing cutinases, for example, from *H. insolens*.^[36]^ Recently, *I. sakaiensis* 201‐F6 was shown to not only degrade PET but also grow on PET as a part of its carbon and energy source.^[36a]^ Two enzymes were detected to be involved in the hydrolysis of the polymer, PETase and MHETase [mono‐(2‐hydroxyethyl)terephthalic acid hydrolase].^[36a]^ Moreover, site‐directed mutagenesis experiments allowed to study the detailed mechanism of PET degradation and to improve the biocatalytic potential of the enzyme.^[37]^ Nonetheless, compared to the mesophilic *Is*PETase, the advantage of thermophilic cutinase in PET degradation has recently been repeatedly emphasized.[^33^, ^38^] In the paper by Tournier et al.,^[33]^ pretreated postconsumer PET waste could almost completely (90 %) be depolymerized by an engineered cutinase within 10 h at a reaction temperature of 72 °C.

For PA, degradation of keratinous proteins in wool by proteases is rather well established.[^23^, ^39^] For synthetic nylons, nylon‐ and nylon‐oligomer hydrolases (E.C. 3.5.1.117) from *Agromyces* sp. have been described.^[40]^ Moreover, three nylon‐oligomer hydrolases from *Flavobacterium* sp. KI72 (6‐aminohexanoate‐cyclic‐dimer hydrolase, 6‐aminohexanoate‐dimer hydrolase, and endotype 6‐aminohexanoate‐oligomer hydrolase) have been reported to hydrolyze cyclic or linear nylon oligomers.^[40]^ Manganese peroxidase (E.C. 1.11.1.13) from *B. adusta* was shown to display activity on Nylon‐6 through an oxidative mechanism, though in low yields.^[41]^ Fungal degradation of Nylon 6,6 was described in 1998, employing the white‐rot fungi IZU‐154, *T. versicolor* and *P. chrysosporium*.^[42]^ Interestingly, all detected products from degradation were related to oxidative attack and no products derived from hydrolysis were found.

Polyurethanes include a high variety of polycarbamates composed of a polyol and a polyisocyanate unit linked by urethane bonds [elastane is formally a poly(urethane‐urea) scaffold]. Polyester‐derived polyurethanes are more susceptible to enzymatic hydrolysis, compared to polyether‐derived structures and in fact many reported ”urethanase” activities can indeed be attributed to the hydrolysis of ester bonds in the soft segment, that is, in the degradation of aliphatic polyester‐polyurethane dispersion (e. g., Impranil DLS from the company Covestro Ltd.).^[43]^ As an example, aryl acylamidase (E.C. 3.5.1.13) from *R. equi* degrades urethane model compounds.^[44]^ As a result of the combination of different types of bonds, the structure is highly unreactive and thus, no enzymatic system has shown to be effective at biodegrading elastane.^[45]^

Degradation of polymers consisting of carbon‐carbon backbones by microorganisms is clearly limited by the lack of hydrolysable functional groups and activating heteroatoms, such as O and N.^[46]^ The non‐hydrolysable bonds in relevant textile polymers, represented here by non‐woven polypropylene used in protective gear in health care and polystyrene used in construction, obstruct their efficient recycling by biocatalysis. Polypropylene can be degraded by oxidative mechanisms to some extent by laccases (EC 1.10.3.2) produced by *P. chrysosporium*.^[47]^ The degradation of polypropylene with microorganisms has been investigated since 1993, which demonstrated that low molecular weight compounds were released only to a low extent.^[48]^ Weight losses up to 18 % were shown in the case of UV pre‐treated samples incubated for one year with two fungal species, *P. chrysosporium* and *E. album*.^[47]^ More recently, an enhanced degradation of up to 55 % weight loss was observed when using a thermophilic consortia of *Brevibacillus* sp. and *Aneurinibacillus* sp. during 140 days.^[49]^ For polystyrene, hydroquinone peroxidase (E.C. 1.11.1.7) from *A. beijerinckii* have shown some biodegradation activity.^[50]^ Mealworms have been shown to depolymerize polystyrene foams^[51]^ assisted by gut microbiota.^[52]^ Lack of identified/isolated biocatalyst prevent their industrial implementation.

## 5 Pre‐Treatment Strategies are Important to Enable High Biocatalytic Activity Facilitating Down‐Stream Processing

Various enzyme‐based approaches have recently been published concerning highly efficient extraction and recovery of monomers (e. g., amino acids or glucose) released from natural fiber fractions (e. g., wool or cellulose) of textile blends.[^24^, ^63^] Mechanical sample preparation and addition of chemical reducing agents to expose reactive bonds in wool is instrumental to achieve high^[23]^ biodegradation activity, pointing towards the importance of pre‐treatment (discussed in more detail below). According to our calculated data (Figure 3A,B), increased rates of 10–10^4^‐fold displayed by existing enzymes are needed for synthetic polymer degradation, in order to reach proficiency at par to the efficiency of cellulose degradation. One potential strategy to achieve this paradigm could be chemoenzymatic pathways, for which a chemical pre‐treatment step^[64]^ (path i in Figure 3C, Table S5, ideally under environmentally benign conditions such as micronization by supercritical fluids) is followed by a biocatalytic step using an engineered enzyme (Figure 3C, path ii). For a heterogeneous system and the hydrolysis of insoluble substrates (i. e., polymers), the adsorption of enzymes on the polymer surface must be taken into consideration.^[64]^ Crystallinity and secondary structure affects the enzymatic rate,[^12e^, ^30^] which is widely known for cellulose degradation and for which cellulose‐binding proteins (including lytic polysaccharide monooxygenases) enhance the enzyme accessibility and thus rate by more than one order of magnitude by pre‐treatment (i. e., oxidative cleavage of biopolymer).^[31]^ The advantage of synergetic chemoenzymatic methods^[65]^ was recently emphasized for proteolysis of natural wool fibers[^23^, ^66^] and by Tournier et al. (2020)^[33]^ in regard to the enzymatic depolymerization of post‐consumer PET bottles. For the latter, a pre‐treatment step involving liquid nitrogen was followed by an enzymatic finishing step using an engineered cutinase, leading to almost full conversion.^[33]^

Association of biocatalyst to synthetic polymers can further be enhanced by fusion to exogenous binding domains.^[12a]^ Enzyme engineering to create matching surface hydrophobicity between polymer and enzyme has been shown for, for example, PET‐degradation^[67]^ and could be expected to be of importance to enhance low enzymatic depolymerization rates of other synthetic materials, including poly(urethane‐ureas). The strong secondary structure interactions and crystallinity render synthetic polymers highly resistant by shielding of reactive bonds (for PET, half‐life >100 years in nature). By utilizing pre‐treatment strategies involving liquid nitrogen to create amorphous PET, enzymatic rate was increased two orders of magnitude^[33]^ (Figure 3C, path i) whereas enzyme engineering applied to plastic degradation applications typically achieves lower rate enhancement (one order of magnitude; Figure 3C, path ii).[^33^, ^54^, ^68^] Hence, a large part of the pre‐requisite biocatalytic rate enhancement can come from enhanced accessibility to the scissile bond, as observed for the rather efficient natural degradation of cellulose.^[55b]^

Increased activity by pre‐treatment is also advantageous for further downstream processing, for example, in yielding elevated amounts of the monomers terephthalate and ethylene glycol (EG) as soluble hydrolysis products from PET. Terephthalate can be readily precipitated by strong acidification to yield terephthalic acid following subsequent filtration and purification,[^33^, ^69^] whereas EG can be recovered by distillation.^[69]^ Obtained monomers can consequently be used in the re‐synthesis of PET with an equivalent quality compared to polymer derived from petrochemical feedstocks,^[33]^ thereby providing opportunities to closing the loop. Alternatively, synthetic‐biology‐based methods enable generation of added‐value products without the need for extraction or purification of released monomers from the product mixture upon biodegradation. Empowering engineered microbes, it was recently shown that terephthalate and/or EG could be valorized to produce other fine chemicals^[70]^ or biopolymers.^[71]^ Utilizing biotechnological solutions together with pre‐treatment could pave the way forward by unlocking a less energy‐consuming biotechnology‐based upcycling strategy of plastic waste compared to traditional chemical approaches.^[12j]^

## 6 Discussion

World fiber demand has increased by 30 Mt from 2010–2020 and grows at a faster pace than the increase in population.^[5]^ This is enough fiber to produce an additional 110 billion garments per year. The growth forecast for textile fibers between 2020 and 2030 is higher than that between 2010 and 2020 (Table S1). Up to now, 3500 Mt of textile fibers have been generated (Table S1, Figure S1, Supporting Information), out of which more than 2500 Mt has been disposed into landfills or incinerated. Approximately half of this quantity has been generated during the last two decades. Since the year 1900, of the estimated 3500 Mt of textile fibers that have been produced, less than 400 Mt of end‐of‐use textile waste has been recycled, with most of this being downcycled. If the current textile production pace persists and novel technology for recycling of fiber blends are not broadly implemented, 6100 Mt of textiles will have been manufactured by 2040, corresponding to an accumulated waste volume of more than 4500 Mt. Reaching a circular textile value chain (herein referred to as “CTVC”) thus requires new business models, careful consideration of resource efficiency, handling of pre‐ and post‐consumer polymer waste, and the development of green processes (Figure 2).

Bio‐based polymers and fibers could be part of the solution for more sustainable textile products, but messages on their environmental and performance benefits remain unclear (water use, land degradation, deforestation, and toxicity from farming chemicals). In fact, the Higg MSI (material sustainability index)^[72]^ scores bio‐based fibers PTT and Nylon 4.10 higher (worse) than fossil‐based equivalents. The LYCRA company launched a bio‐based elastane in 2014, but this failed to gain market traction, mostly due to cost and concerns over competition for land use/food production. Biodegradability has been associated with bio‐based synthetic fibers, but this is not the case with all polymers.^[12c]^ Bio‐based PET and elastane have the same properties as their fossil‐derived equivalents.^[15]^ PLA biodegrades^[73]^ but does not meet compost standards, while it can be processed through industrial composting facilities. Biodegradability and composting is not seen as a preferred route for end‐of‐use textile waste and is increasingly challenged as waste does not biodegrade efficiently in landfills.^[1c]^

The challenge and systemic change required to develop innovative recycling solutions is perhaps well illustrated by current low availability of high‐quality sorted fiber fractions, corresponding to only 0.49 Mt y^−1^ for Northwestern Europe^[17]^ and up to 11 Mt y^−1^ globally. Moreover, using elastane as an example, lack of recycling technology currently leads to destruction of 0.1 Mt y^−1^ of already available, collected, and potentially valuable recyclable material in Northwestern Europe^[17]^ and around 2 Mt globally,^[13]^ only by the presence of this polymer type. Warp knit has the highest elastane content with 15–30 % (swimwear, shapewear, etc.), followed by circular knit/jersey (mostly 8–12 %), ladies hosiery (5–20 %), and finally wovens (2–5 %). Assuming an elastane average blend of 90 : 10, spandex is today partnering with around 8 Mt of other fibers. With a ratio of 95 : 5, then the other fibers blended with spandex would be around 16 Mt (estimate). These numbers are projected to grow significantly by 2030 (Tables S1 and S2), which stresses the need for efficient recycling technologies.

Part of the challenge in recycling textile waste is to be able to regenerate virgin‐quality equivalent materials at an affordable cost and with a minimum environmental footprint. Thermomechanical separation of blends composed of elastane and PA herein showed that both techniques could handle low amounts of elastane by down‐cycling but failed to handle feedstock containing higher amounts (Tables S6 and S7). Drying of the material is instrumental to avoid degradation caused by absorbed water and longer drying times resulted in improved mechanical properties of generated test bars upon injection molding. Washing of pre‐consumer fabric is vital to get rid of processing aids (such as spin oil, additives, etc.) and to improve mechanical properties of generated materials. If the processing aids are not removed, they affect down‐stream processing (injection molding, melt spinning). Depending on content, some materials are better used today as an energy source due to non‐compliance or difficulties in achieving perceived quality. Some materials will have a value that matches other end uses, such as insulation, fibers in construction materials, and materials to be used in plastic parts, sometimes referred to as downcycling, and is more about extending life than being part of the CTVC.

The level of maturity of different possible separation methods differs and is also material dependent. Some require pure materials and others show promising results for material blends,^[74]^ in particular biocatalysis that could achieve specific depolymerization of individual components. Aiming at raw material and monomer recovery within the context of a circular economy, it is encouraging to see that enzymatic depolymerization of PET has successfully been accelerated by 2–3 orders of magnitude over the last decade.[^12h^, ^37b^, ^54^, ^75^] Although a biocatalytic process aiming at preferential degradation of the synthetic polyester fraction in complex fabric blends has not yet been described, enzymatic depolymerization of textiles with high polyester content was recently achieved.^[25]^

Most likely, several recycling steps need to be connected in a value chain as shown in Figure 2 in order to create a valuable secondary raw material enabling new textile material production. It is envisaged that going from a linear system to create a true circular system and economy for textiles, the value chain has to change, and new technologies have to be adapted to handle complex blends such as materials containing elastane. Accounting for the dilution of elastane by its utilization as a minority blend fiber, it is estimated that, albeit its total small quantity, elastane currently renders approximately 16 Mt of textile that is difficult to recycle; a number equivalent to around 4000 trucks per day of merchandise and that could grow to 50 Mt by 2030. This highlights the complexity of blended fibers associated with modern fashion, a trend that, when exemplified for elastane alone, has contributed to the accumulation of 300 Mt of non‐recyclable waste between 2000–2020; a number projected to grow to 500 Mt by 2030.

## 7 Summary and Outlook

Chemical separation technologies of textile waste are under development[^64^, ^76^] and may be designed in the future to handle high concentrations of contaminants such as elastane during processing. As shown in Figure 3, enzymes have been reported for some polymer types of relevance to the textile industry. By the data analysis herein, it is shown that only enzymatic polyethylene terephthalate (PET) hydrolysis is at par to cellulose hydrolysis, and this has been recently shown to reach industrial‐implementation potential for pre‐treated pure PET packaging waste.^[33]^ Analogously, various pre‐treatment approaches trivially used for textile wastes should be evaluated in the future towards amorphized polyester fibers fulfilling the low crystallinity required for rapid enzymatic depolymerization. As polyester (PET)‐cotton blends are the most abundant fabrics blends used in the textile industry, which will consequently end up as textile waste difficult to be separated, using multiple enzyme catalysts containing a “cocktail” of both industry‐relevant cellulases and PET hydrolase may hold great promise for future development of a biocatalytic recycling process beyond pure textile materials.^[25]^ More precisely, the anaerobic bacterium *Clostridium thermocellum*, which can intrinsically degrade cellulose using a highly effective cellulosome as a multienzyme complex of a wide range of secreted polysaccharide‐hydrolyzing enzymes, has been recently engineered to catalyze PET hydrolysis by expressing a recombinant PET hydrolase.^[77]^ In this way, a future development of a whole‐cell based degrader of polyester (PET)‐cotton blends fiber waste could be within reach. It is encouraging to see that polyurethanases capable of cleaving the reactive nitrogen atom of the urethane bond have been reported.^[57c]^ One potential route would be to recover the majority fiber in the blend by enzyme catalysis[^24b^, ^63^, ^78^] (nylon, polyester, cotton, etc.), and to leave the minority fiber (e. g., elastane) as part of the residue to be recycled via thermochemical processes or gasified for energy recovery. Based on our data, we advocate backcasting fashion towards materials with a cotton base: a combination of well‐established cellulase degradation of cellulose‐based fibers together with plastic‐based fiber degradation would be the central future target for circular textiles.

## Experimental Section

### Data collection

Data were collected via literature search and via industry dialogue. Industries participating in the study, providing insights on material flow and detailed data for recycling technologies, were mainly raw material producers of synthetic virgin and secondary raw materials, as well as sorting facilities. Survey details are given in the Supporting Information. Data for past and predicted future fiber demand was compiled from data from industrial partners and from literature study.

### Recycling study

Two different separation routes (melt filtration and rotor spinning) were used to investigate if it was possible to recycle PA/elastane blends for further processing towards a secondary raw material.

**Melt filtration followed by melt spinning**: Trials have been performed as follows with the following material specifications (detailed list shown in Supporting Table S6, equipment specified in Table 1):

**Table 1:** Specifications of equipment for separation study.

Method	ISO‐standard	Manufacturer	Model	Type of equipment
compounder/extruder	–	Coperion	ZSK 26K 10.6	–
melt filtration equipment	–	Gneuss	HSM 45	–
melt filters	–	Gneuss	different sizes	filter
injection molder	injection moulding of test bars according to ISO 527‐2 Type 1 A	Engel	ES 200/110 HL‐V	–
tensile test	ISO 527–2	MTS	20‐M	universal tensile testing equipment
charpy impact	ISO 179	Instron	Ceast 9050	notching equipment
charpy impact	ISO 179	Instron	Ceast AN50	impact toughness tester
density	ISO 1183‐A	Mettler Toledo	AT200	scale
textile tearing equipment	–	New Shun Xing	NSX‐QT310	–
carding machine	–	Cormatex	CC/400	–
drawing frame	–	Suessen	Stiro Roving Lab	–

- model system of virgin PA6 (Ultramid® B24N 03) mixed with 0–15 % elastane (Roica BX)
- ground fabric: post‐industrial (PA6 mixed with 22 % elastane)
- ground pantyhose (PA6.6 mixed with ≈10 % elastane)
- ground fabric: post‐industrial (PA6 mixed with ≈8 % elastane)

Different filter sizes were used (20, 25, and 60 μm), where 25 μm is the finest commercially available filter. The 20 μm filter was custom built in‐house. Different temperature settings have been used, depending on material (PA6 melts at ≈215 °C and PA6.6 at ≈267 °C). The temperature profile for the twin screw extruder with melt filter attached varies between the different zones; the material needs to be melted when it goes through the filter, otherwise it will get stuck. The temperature has, however, been held as low as possible to decrease degradation of both the elastane and polyamide, resulting in very low feeding speed and thereby longer residence time. Regarding mechanical properties, polyamide mixed with elastane performs like an impact‐modified polyamide grade. No successful trials with melt spinning when using commercial equipment were achieved. Trials were made with both the model system grades and with recycled materials mixed with virgin polyamide (Table S6, Supporting Information). As an alternative route, injection molding was used to generate test bars that were further characterized according to ISO 527‐2, ISO 179, and ISO 1183‐A (Table S7, Supporting Information).

**Rotor spinning**: The following material sets were tested, using equipment specified in Table 1:

- 78 % PA6.6/22 % elastane
- 93 % PA6/7 % elastane
- 94 % PA6.6/6 % elastane
- 95 % PA6.6/5 % elastane

First the materials were cut manually into smaller pieces, thereafter they were fed through the textile tearing machine. Unfortunately, the material containing 22 % elastane disappeared in the machine during tearing (probably due to the high elastane content). After tearing, a sliver is produced in a drawing frame, and the quality of the sliver has a large impact on the quality of the yarn. When producing the sliver, the fibers get drawn out and mixed, and the amount of fibers is reduced. The reason is to achieve the desired density, as well as to increase uniformity for the rotor spinning procedure to start. To facilitate the rotor spinning process, the recycled fabric was mixed with another textile material; in this case a mixture of 20 % polyester and 80 % recycled fabric was used.

### Calculation of estimated enzymatic rates of depolymerization

For polypropylene, laccase‐mediated depolymerization data was taken from ref. [58] for which polyethylene was used as substrate. 20 % conversion of the carbon backbone was achieved after composting at 37 °C for 80 days. For polystyrene, rates of polymer consumption were based on data from ref. [51a]. Specifically, a conversion of 67 % after 31 days at 25 °C by the mealworm *T. obscurus* was reported.^[51a]^ Polymer decomposition in the gut of mealworm was demonstrated.^[51a]^

For polyurethane degradation, data for growth experiments of fungi utilizing polyester‐derived polyurethanes as carbon source was taken from ref. [57a]. Kinetic data using purified enzyme at room temperature was directly taken from ref. [57b].

For wool, conversion data was taken from ref. [23]. Specifically, a weight loss of 99.5 % was observed after proteolysis for 15 h at 50 °C.

For the purified nylon hydrolase from *Agromyces sp*, the enzymatic rate and polymer conversion data were based on the formation of 3 mm amine product after 5 h from PA6.6 utilizing 0.1 mg mL^−1^ enzyme at 60 °C.^[56]^ Data on polymer conversion was compared to that of degradation of PA6 fibers by microbial depolymerization by *B. adusta*.^[41]^ The nitrogen content in the filtrated sample was assumed to originate from enzymatic oxidative degradation by manganese peroxidase.

Kinetics and conversion data for *H. insolens* cutinase‐catalyzed depolymerization of PET was taken from ref. [36b]. The highest activity was 6.1 μmol mL^−1^ h^−1^ at 70 °C at an enzyme concentration of 6 nmol mL^−1^. Two titratable groups in terephthalic acid were assumed. Conversion data for pre‐treated, bottle‐grade PET was taken from ref. [33].

Kinetic data for hydrolysis of high‐crystalline PET by an engineered variant of *I. sakaiensis* PETase was taken from ref. [55c]. Specifically, 1.8 mm of building blocks were generated in 72 h at a temperature of 40 °C. The enzyme concentration was assumed to be 0.01 mg mL^−1^. For PET nanoparticles, 1 mm of monomeric products were generated in 1 h at 37 °C utilizing an enzyme concentration of 0.002 mg mL^−1^.

For hydrolysis and depolymerization of cellulose, conversion data was taken from ref. [55b] for which northern bleached softwood Kraft cellulose fibers were utilized. A conversion of 30 % with respect to released monosaccharide was reported after 6 h.^[55b]^ For crystalline β‐chitin, experimental data was taken from ref. [31]. Addition of lytic polysaccharide monooxygenase yielded a conversion of 57 % after 2 h, calculated based on the detected dimeric building block [i. e., (GlcNac)_2_, *M*_w_=424.4 g mol^−1^]. The absolute rate for chitin depolymerization was calculated to be 500 h^−1^ based on data in ref. [31]. Rate constants for hydrolysis of phosphoric acid swollen cellulose by *H. insolens* cellulases were taken from ref. [55d] (average rate constant was calculated to be 32 s^−1^ at pH 8.5).

## Competing interests

During the execution of this work, Andreea Toca was an employee of Swedish Stockings, Tyskbagargatan 7, 114 43 Stockholm, Sweden.

Les M. Jacques is an employee of The LYCRA Company UK Limited, 60, Clooney Road, Maydown, Londonderry, N. Ireland. BT47 6TH, UK.

## Biographical Information

*Christina Jönsson studied Organic synthesis and received her PhD at Royal Institute of Technology (KTH), Stockholm in 2003. With focus on analytical chemistry and surface chemistry she did a two‐year industry post doc at Dublin City University (DCU) in Dublin and the Swedish based bio diagnostic company Åmic on a Marie Curie scholarship. Since 2009, she has worked at RISE as a researcher in the field of chemical management, traceability and transparency and life cycle assessment. With expertise in sustainability, she has performed commissions and research project together with academic partners, industry, and authorities with focus on activities in the textile value chain. Currently she is vice president for the department Product realization methodology within the division of Material and Production*.

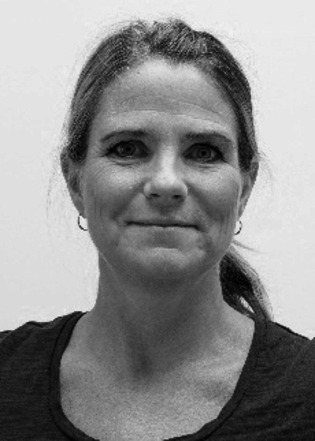

## Biographical Information

*Uwe T. Bornscheuer studied chemistry and received his PhD in 1993 at Hannover University followed by a postdoc at Nagoya University (Japan). In 1998, he completed his Habilitation at Stuttgart University about the use of lipases and esterases in organic synthesis. He has been Professor at the Institute of Biochemistry at Greifswald University since 1999. Beside other awards, he received in 2008 the BioCat2008 Award. He was just recognized as ‘Chemistry Europe Fellow’. His current research interest focuses on the discovery and engineering of enzymes from various classes for applications in organic synthesis, lipid modification, degradation of complex marine polysaccharides and plastics*.

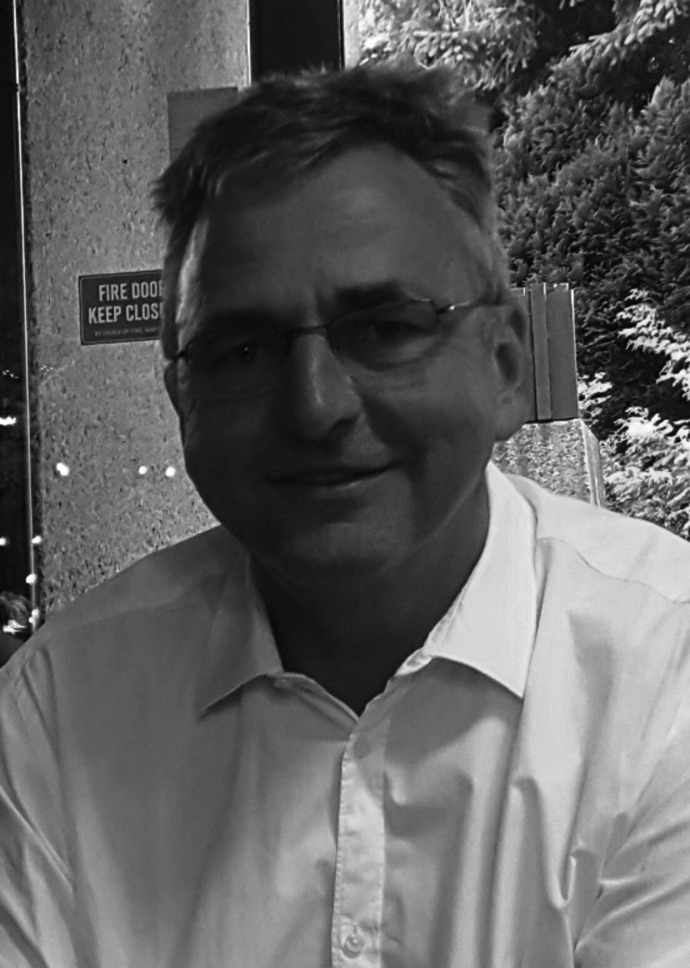

## Biographical Information

*Per‐Olof Syrén obtained his PhD in biotechnology from KTH in Sweden in 2011. After a postdoc at the Institute of Technical Biochemistry in Stuttgart as an Alexander von Humboldt Fellow, he returned to KTH to establish his independent research group. His research combines biotechnology, biocatalysis, enzyme design and polymer chemistry with the overarching goal to contribute to a better environment and health. In 2019, he was awarded the competence development award from his Majesty King Carl XVI Gustaf of Sweden and the Gunnar Sundblad Research Foundation for his work on polymer retrobiosynthesis. He is Associate Professor in Chemistry since 2016*.

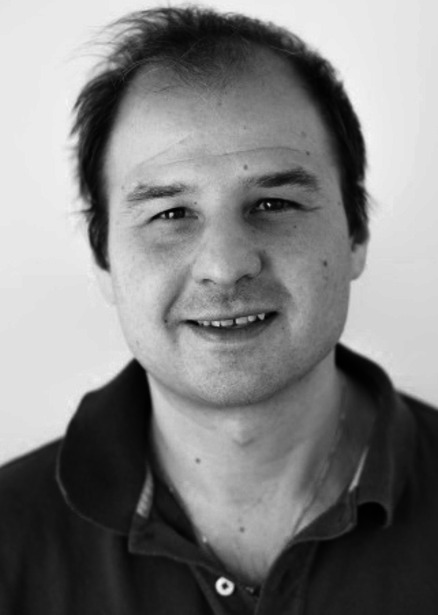

## Supporting information

As a service to our authors and readers, this journal provides supporting information supplied by the authors. Such materials are peer reviewed and may be re‐organized for online delivery, but are not copy‐edited or typeset. Technical support issues arising from supporting information (other than missing files) should be addressed to the authors.

Supporting Information
