# Supplementary material for: Biocatalysis in the Recycling Landscape for Synthetic Polymers and Plastics towards Circular Textiles
Source: ChemSusChem. 2021 Feb 12;14(19):4028–40. doi: 10.1002/cssc.202002666 (PMC8518944; doi:10.1002/cssc.202002666)
Supplement: Supplementary file 1 — Supporting Information [file CSSC-14-4028-s001.pdf]

ChemSusChem

Supporting Information

Biocatalysis in the Recycling Landscape for Synthetic Polymers and Plastics towards Circular Textiles

Christina Jönsson,* Ren Wei, Antonino Biundo, Johan Landberg, Lisa Schwarz Bour, Fabio Pezzotti, Andreea Toca, Les M. Jacques, Uwe T. Bornscheuer,* and Per-Olof Syrén*This publication is part of a collection of invited contributions focusing on "Chemical Upcycling of Waste Plastics". Please visit to view all contributions.© 2021 The Authors. ChemSusChem published by Wiley-VCH GmbH. This is an open access article under the terms of the Creative Commons Attribution License, which permits use, distribution and reproduction in any medium, provided the original work is properly cited.

Table S1. Global textile mill consumption (Mt/year). Data adapted from reference[1].

Fiber type	2000	2010	2015	2020	2025	2030
Wool	1.455	1.117	1.132	1.122	1.153	1.176
Cotton	19.97	24.692	23.774	26.117	28.514	30.792
Cellulose-based fibers	2.292	3.605	5.375	6.509	8.658	10.408
Acrylic	2.637	1.914	1.793	1.677	1.686	1.679
Nylon	4.044	3.688	4.107	5.051	5.739	6.302
Polyester	19.345	37.088	47.783	59.02	69.81	82.123
Polypropylene	2.916	3.58	4.262	5.332	6.336	7.582
Elastane	0.174	0.414	0.619	0.845	1.125	1.4
Total	52.659	75.684	88.226	104.828	121.896	140.062

Table S2. Market share for different fiber types in textile applications [%].

Fiber type	2000	2010	2015	2020	2025	2030
Wool	2.8	1.5	1.3	1.1	0.9	0.8
Cotton	37.9	32.6	26.9	24.9	23.4	22.0
Cellulose-based fibers	4.4	4.8	6.1	6.2	7.1	7.4
Acrylic	5.0	2.5	2.0	1.6	1.4	1.2
Nylon	7.7	4.9	4.7	4.8	4.7	4.5
Polyester	36.7	49.0	54.2	56.3	57.3	58.6
Polypropylene	5.5	4.7	4.8	5.1	5.2	5.4
Elastane[a]	0.3	0.5	0.7	0.8	0.9	1.0
Total	100.0	100.0	100.0	100.0	100.0	100.0

[a] Asia has become dominant in mill consumption of elastane (as with other fibers). In 2000, Asia's market share was 48%, Greater Europe's was 24% and the America's was 28%. In 2020, Asia's forecasted market share will be 83%, Greater Europe's will be 9.5% and the America's will be 8.5%.[1]

Table S3. Generic overview regarding fiber categories, polymer blends and their use in different applications. The table shows some common fiber blends with an emphasis on polymers with hydrolysable links, relative abundancy (%) of each fiber type and in what application they may be found. The data is synthesized from several different sources, i.e. industry dialogue and research projects, and only give a simplified overview over the complex textile material flow. However, the data give a basic understanding of the need for separation technologies enabling high value textile secondary raw material.

**Fiber category**	**Fiber type**	**Common product type**
Cellulosic	Cotton	T-shirts, sweatshirts, bed linen
Regenerated cellulose	Viscose	T-shirts and tops, carpets
	Lyocell	T-shirts, bed linen
Synthetic fiber	Polyester	Workwear, home textile, lining, shell
	Polyamide 6	Technical textile, rainwear, sunscreens, outdoor (lining, shell)
	Polyamide 6,6	Technical applications
	Polyurethane	Synthetic leather articles and accessories
Natural fiber	Wool	Socks, under clothing
Cellulose-based/synthetic blends	Cotton/polyester (polycotton)	Workwear, health care textile, bedsheets, sweatshirts
	Polyester/elastane (lower elastane content, 3-15%)	Tops, dresses, trousers
	Polyamide/elastane (usually higher elastane content, 10-25%)	Swimwear, sportswear
	Cotton/elastane (lower elastane content, 1-5 %)	Denim, t-shirts, sweatshirts
	Viscose/elastane (lower elastane content)	T-shirts, trousers
	Viscose/polyamide	T-shirts, tops
	Wool/polyamide	Knitwear, sportswear
	Wool/polyamide/elastane	Knitwear, sportswear

Table S4. World fiber demand since 1900. Data adapted from references [1]-[2].

Year	Total amount of fibers produced [Mt]
1900	4
1910	6
1920	7
1930	8
1940	10
1950	13
1960	20
1970	25
1980	30
1990	41
2000	53
2010	76
2015	88
2020	105
2025	122
2030	140

Table S5. Some existing methods for separation of complex textile materials and pre-treatment of plastics and polymers.[3]

Technology	Description
Mechanical recycling	
Mechanical processing	Removal of buttons, zippers and other macrostructural elements.<br><br>Shredding, milling, grinding and sieving), depending on method for further processing, to generate fiber or smaller particles.
Thermomechanical	Separation of components based on difference in melting temperatures.<br><br>Enables compounding and injection molding.
Chemical	
Dissolution	Separation and removal of components that could interfere with recycling (e.g. adhesives, dyes).<br><br>Chemical micronization/restructuring by supercritical fluids to enhance accessibility of polymer chains.
Depolymerization (e.g. by acid)	Generation of shorter polymer fragments, oligomers and finally monomers.

Supplementary note on melt filtration

Table S6. Material specifications and experimental setup.

Material nr.	Material type	Polyamide	Amount of Elastane [%]	Additive	Washed	Drying before compounding[a]	Drying before injection molding[a]	Melt filtration	Comment
1	Reference	Ultramid® 8202 HS (PA6 - virgin)	-	-	-	-	-	-	-
2	Reference	Ultramid® 8253 HS (PA6 - virgin)	-	-	-	-	-	-	
3	Model system	PA6 (virgin)	0		-	72 h	72 h	No	-
4	Model system	PA6 (virgin)	5		-	72 h	72 h	No	-
5	Model system	PA6 (virgin)	10		-	72 h	72 h	No	-
6	Model system	PA6 (virgin)	15		-	72 h	72 h	No	-
7	Model system	PA6 (virgin)	5	-	-	24 h	72 h	Yes	-
8	Model system	PA6 (virgin)	10	-	-	24 h	72 h	Yes	-
9	Consumer fabric	PA6 (post-industrial)	8	-	No	24 h	48 h	Yes	Very problematic during IM[b]
10	Consumer fabric	PA6 (post-industrial)	8	-	5 times at 40 °C	24 h	48 h	No	Worked well during IM
11	Consumer fabric	PA6 (post-industrial)	8	-	5 times at 40 °C	24 h	48 h	Yes	Worked well during IM
12	Consumer fabric	PA6 (post-industrial)	22	-	No	72 h	168 h (initial trials after 72 h failed)	No	-
13	Reference	Ultramid® A3K BK00464 (PA6.6 - virgin)	-	-	-	-	-	-	
14	Reference	Ultramid® A3K R01 (PA6.6 - virgin)	-	-	-	-	-	-	
15	Pantyhose	PA6.6	10	-	No	72 h	72 h	Not possible	Minor issues during IM
16	Pantyhose	PA6.6	10	-	No	4 h	72 h	Not possible	Minor issues during IM
17	Pantyhose	PA6.6	10	-	No	4 h	4 h	Not possible	Problematic during IM
18	Pantyhose	PA6.6	10	-	No	72 h	4 h	Not possible	Problematic during IM
19	Pantyhose	PA6.6	10	0.5 % Licomont CaV	No	24 h	24 h	Not possible	Worked well during IM
20	Pantyhose	PA6.6	10	0.1 % Addworks	No	24 h	24 h	Not possible	Worked well during IM

[a] Drying temperature 80 °C. [b] IM = injection molding.

Table S7. Mechanical properties of the different test bars generated (conditions from Table S6).

Material nr.	Elastic modulus [MPa]	Stress at yield [MPa]	Strain at yield [%]	Stress at break [MPa]	Strain at break [%]	Impact resistance, Charpy [kJ/m²]	Density [g/cm³]
**Method**	ISO 527-2	ISO 527-2	ISO 527-2	ISO 527-2	ISO 527-2	ISO 179	ISO 1183-A
1	2700	78	4	-	25	3.5	1.13
2	2300	60	4	-	40	18	1.09
3	2631±84	77.9±3.1	4.6±0.7	65.9±15.7	16.5±17.6	-	1.13
4	2586±53	72.3±0.2	5.5±0.1	65.5±2.5	23.1±6.1	-	1.12
5	2361±51	65.0±0.3	5.1±0.1	57.5±0.6	25.3±1.2	-	1.12
6	2233±70	58.7±0.2	4.8±0.1	51.5±1.7	17.3±3.4	-	1.11
7	2695±44	62.7±0.9	3.9±0.1	49.9±9.8	20.3±9.8	4.8±0.1	1.12
8	2562±31	57.2±0.5	3.7±0.0	37.3±13.2	44.0±19.8	5.2±0.3	1.12
9	3015±148	-	-	48.8±8.4	2.1±0.9		-
10	3140±94	75.4±0.4	3.8±0.1	56.8±1.2	13.5±0.2	-	-
11	3120±82	-	-	52.9±19.8	2.0±1.0	-	-
12	2157±41	49.8±0.2	3.9±0.0	39.2±3.9	22.6±5.6	-	-
13	3600	87	4.2	-	-	5	1.13
14	3000	85	5	-	20	5	1.14
15	2912±57	-	-	44.0±4.4	1.6±0.2	2.1±0.6	1.13
16	2913±83	-	-	37.8±1.6	1.4±0.1	2.2±0.6	1.14
17	3030±39	-	-	42.2±4.2	1.6±0.2	1.4±0.1	-
18	3207±96	-	-	32.7±3.5	1.1±0.1	2.1±0.6	-
19	3203±115	-	-	45.3±9.1	1.5±0.4	2.5±0.6	-
20	3164±73	-	-	45.9±10.0	1.6±0.4	2.6±0.4	-

Brief summary of results obtained:

Comparing material 10-12 (Table S6), the importance of washing to remove processing aids is seen. The washed and unfiltered material shows significantly improved mechanical properties than the other two (Table S7), while both materials that had undergone washing had superior processing properties during injection molding.

As seen in Table S6 and S7, comparing properties for materials nr. 15-18, drying before injection molding seems more important than before compounding (both regarding mechanical properties of test bars obtained and process properties during injection molding). By adding additives (Table S6, bottom), the material properties improved greatly during the injection molding process, while the mechanical properties showed a slight improvement (Table S7).

Comparing to the respective virgin PA-grades, the washed and unfiltered consumer fabric (nr. 10) and the two pantyhose materials with additives (nr. 19 and 20) shows promising properties, both in terms of mechanical properties of test bars and processability.

Supplementary note on rotor spinning

The following results were obtained:

- 78 % PA6.6 – 22 % Elastane: The material disappeared in the textile tearing machine
- 93 % PA6 – 7 % Elastane: Too high abundancy of contaminants including elastane to be able to rotor spin (twisted threads from other fabrics and elastane, very short fibers)
- 94 % PA6.6 – 6 % Elastane: The fibers were too long

- 95 % PA6.6 – 5 % Elastane: The fibers were long, but a short thread could be rotor spun. During the rotor spinning process one could see that part of the elastane was separated.

Table S8. Physiochemical properties of synthetic textile fibers shown in Figure 1, inherent reactivity of relevant chemical bonds and their susceptibility to enzymatic degradation (given as % conversion of polymer s^{-1} and/or as rate-constants calculated from published experimental data). Experimentally determined bond dissociation energies and half-life of uncatalyzed hydrolysis of the scissile bond reflects monomeric/oligomeric substrate.

**Fiber component**	T_g (° C)	**Scissile bond**[a]	**Available biocatalysts/ Biotechnological system**	**Rate**[b]	**References**
		Carbon-carbon bonds			
Polypropylene	-20 to 0 [c],[4]	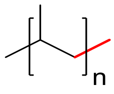 **89**	*Phanerochaete chrysosporium* Laccase, *Engyodontium album* Laccase, *Pseudomonas* sp. Alkane hydroxylase	10^{-6} % s^{-1} [d]	[5],[6]
Polystyrene	95 [4]	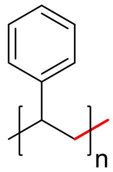 **77**	*Azotobacter beijerinckii* Hydroquinone peroxidase, Mealworms (*Tenebrio molitor, Tenebrio obscurus*)	10^{-5} % s^{-1} [e]	[7]
		Carbamates			
Polyurethane	-51 to -57 [f],[8]	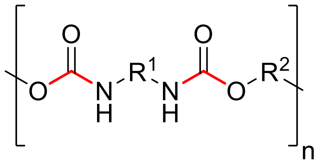 **$t_{1/2}$ = 183 000 years** (breaking of ester bond and formation of isocyanate)	Fungal/bacterial polyurethanases	10^{-5} % s^{-1}[g], (0.01 s^{-1} [h])	[9],[10]
		Ureas			
Urea	-	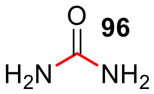 **96** **$t_{1/2}$ = 1800 years**	Ureases	3500 s^{-1} [i]	[11]
		Polyamides			
Polyamide (Nylon)	53 (nylon 6), 57 (nylon 6,6)[12]	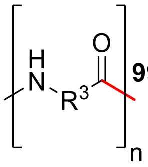 **99** **$t_{1/2}$ = 400 years**	*Agromyces sp.* Nylon hydrolase, *Bjerkandera. adusta* Manganese peroxidase	10^{-4} [j] - 10^{-6} [k] % s^{-1} (0.06 s^{-1} [l])	[13],[14]

Polyesters					
Polyester (PET)	75 [15]	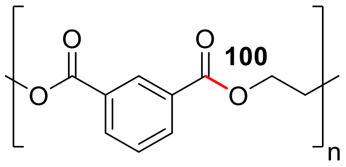 $t_{1/2}$ = 4 years	PETases, Cutinases/poly-esterases	10^{-2} % s^{-1} [l], 0.1 s^{1} (0.004 s^{-1})[m], 4 s^{-1} (0.02 s^{-1}) [n]	[16]
Polysaccharides					
Cotton	200–250 [17],[o]	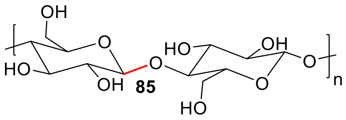 $t_{1/2}$ = 5*10^{6} years	Glycoside hydrolases	10^{-3} % s^{-1} [p] - 10^{-2} % s^{-1} [q] (corresponding to 0.1 s^{-1})([q] (Pre-treated cellulose, 32 s^{-1})	[18] [19],[16h, 20]

[a] Scissile bond high-lighted in red with bond dissociation energy[21] given in kcal/mol. For cotton, the bond dissociation energy is represented by a glycosidic ether bond due to lack of experimental data. Experimental data for carbamates was not available as well. Half-life of uncatalyzed hydrolysis reactions are given at 25 °C for the relevant scissile bond [22] (excluding polyolefins for which hydrolysis mechanisms do not operate). [b] Given as conversion of polymeric substrate to monomers or metabolized products. Rate constants are given within brackets, where accessible from experimental data. See Materials and methods for details. [c] Depending on the tacticity. [d] Data for composting of polyethylene at 37 °C. [e] Consumption at 25 °C analyzed by weight loss, depolymerization in insect gut demonstrated. [f] Depending on the chain extender units. [g] When using polyester polyurethane as a sole carbon source to sustain fungal growth (at 25 °C). [h] Data for purified protein at room temperature. [i] Data for monomeric urea and utilizing purified protein at 37 °C. [j] Data for degradation of powdered nylon 6,6 using purified nylon hydrolase (data given at 60 °C). [k] Data for degradation of Nylon 6 fibers by *B. adusta* at 30 °C. [l] Depolymerization of amorphous nanoparticles at 37 °C. [m] For depolymerization of low-crystalline PET catalyzed by *H. insolens* cutinase at 70 °C. Activity for PET with 35% crystallinity were two-orders of magnitude lower and given within brackets. [n] Kinetic data for semi-crystalline PET depolymerized by an engineered variant of *I. sakaiensis* PETase at 37 °C.[16h] Corresponding data for high-crystalline PET is given in brackets (at a temperature of 40 °C). [o] Depending on the actual cellulose-based polymer, its composition in terms of crystallinity index and degree of polymerization. [p] Data for degradation of northern bleached softwood Kraft cellulose fibers given at 50 °C. [q] Data for degradation of crystalline β-chitin at 37 °C.

Supplementary Note on Enzymatic conversions

It is interesting to note that conversion of more resilient polymers, including polyolefins and polyamides, converge to polymer conversions of around 10^{-4}-10^{-6} % s^{-1}, independent of the system under study (Table S8). There is no clear relationship between bond dissociation energies and half-life associated with uncatalyzed hydrolysis of the relevant bond type; a fact which is perhaps well illustrated by the aromatic ester bond (Table S8).

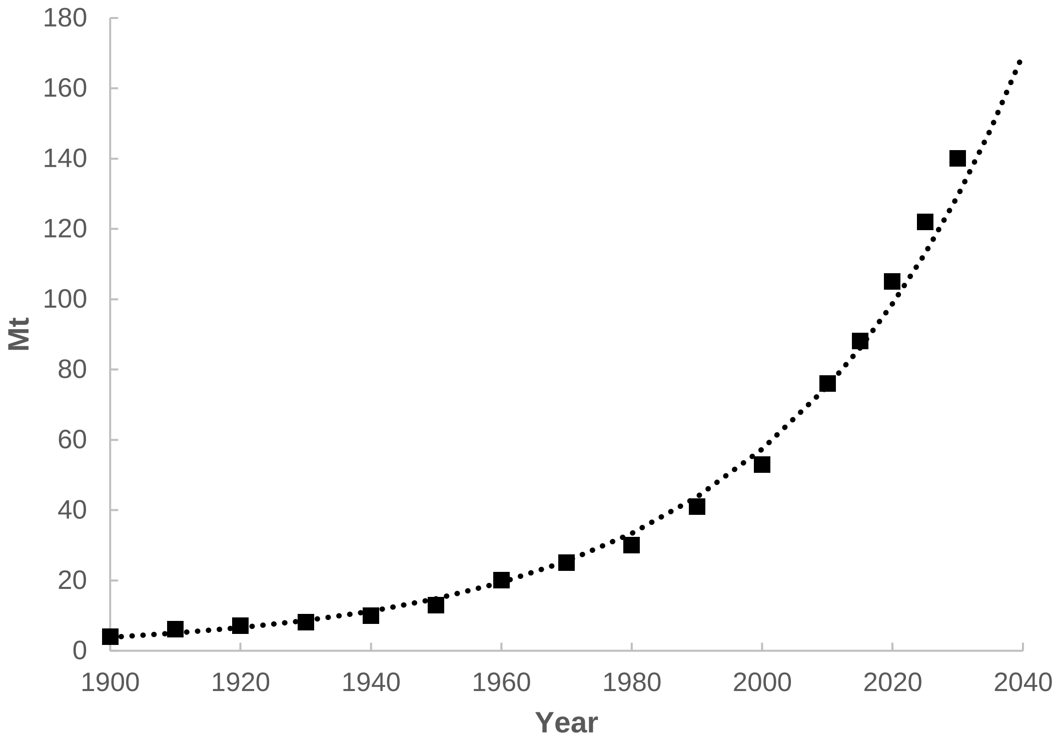

Figure S1. World fiber demand since 1900 and future prognosis under a business as usual scenario. Data adapted from references [1]-[2] and based on information provided by industrial partners. The dotted line corresponds to fitting of the data to an exponential function using non-linear regression, in order to calculate total production volumes by integration. $y = 1.6295*10^{-22}*e^{0.027109x}$, $R^2 = 0.9944$.

Data collection and Survey

Questions were exposed via a Google survey to Nordic textile brands via the Swedish Chemicals Group at RISE†. The number of answers were about 20 and an overview of the questionnaire including a summary of the answers is presented below.

Question	Summary of answers
"Do you use any fiber blends with elastane content?"	75% of incoming answers was yes
"If yes, what kind of mix (e.g. polyester with elastane)?"	Polyester, PA, Wool, Cotton
"And in what percentage of elastane?"	The range of percentage of elastane according to the questionnaire is between 2-30%. The higher end of the range of elastane (approx. 20%) content is used in polyamide fabrics, for example swim wear. In the lower range it was stated that polyester-elastane mixtures were most common.
"Do you source any recycled material?"	All incoming answers was yes
"What type of recycled fibers do you source?"	Both pre- and post- consumer waste. Mainly food packaging material (e.g. PET bottles)
"Are the materials certified?"	All incoming answers was yes:<br>By GRS or U TRUST certificate (REPREVE)
"Do you recycle any production waste, i.e., pre consumer?",	60% of the incoming answers was yes
"If yes, what material?"	Cotton, in one case PA

† https://www.ri.se/en/what-we-do/networks/chemicals-group

Supplementary References

[1] Textile Fibres Global Supply Demand Forecast to 2030. **2019**. Wood Mackenzie.

[2] J. Mills, in *Tecnon OrbiChem presentation at Association Française Cotonnière (AFCOT), Deauville, France, 6 October 2011.*, **2011**.

[3] I I. Vollmer, M. J. F. Jenks, M. C. P. Roelands, R. J. White, T. van Harmelen, P. de Wild, G. P. van der Laan, F. Meirer, J. T. F. Keurentjes, B. M. Weckhuysen, *Angew. Chem., Int. Ed.* **2020**, *59*, 15402-15423.

[4] C. E. Wilkes, J. W. Summers, C. A. Daniels, M. T. Berard, *PVC Handbook*, Hanser, **2005**.

[5] D. Jeyakumar, J. Chirsteen, M. Doble, *Bioresour. Technol.* **2013**, *148*, 78-85.

[6] M. G. Yoon, H. J. Jeon, M. N. Kim, *J. Biorem. Biodegrad.* **2012**, *3*, 145.

[7] a) K. Nakamiya, G. Sakasita, T. Ooi, S. Kinoshita, *J. Ferment. Bioeng.* **1997**, *84*, 480-482; b) S. S. Yang, A. M. Brandon, D. F. Xing, J. Yang, J. W. Pang, C. S. Criddle, N. Q. Ren, W. M. Wu, *IOP Conference Series: Earth and Environmental Science* **2018**, *150*; c) B. Y. Peng, Y. Su, Z. Chen, J. Chen, X. Zhou, M. E. Benbow, C. S. Criddle, W. M. Wu, Y. Zhang, *Environ. Sci. Technol.* **2019**, *53*, 5256-5265.

[8] E. Orgilés-Calpena, F. Arán-Aís, A. M. Torró-Palau, C. Orgilés-Barceló, *Prog. Rubber. Plast. Re.* **2011**, *27*, 145-160

[9] J. R. Russell, J. Huang, P. Anand, K. Kucera, A. G. Sandoval, K. W. Dantzler, D. Hickman, J. Jee, F. M. Kimovec, D. Koppstein, D. H. Marks, P. A. Mittermiller, S. J. Núñez, M. Santiago, M. A. Townes, M. Vishnevetsky, N. E. Williams, M. P. N. Vargas, L.-A. Boulanger, C. Bascom-Slack, S. A. Strobel, *Appl. Environ. Microbiol.* **2011**, *77*, 6076-6084.

[10] a) J. Schmidt, R. Wei, T. Oeser, L. A. Dedavid E Silva, D. Breite, A. Schulze, W. Zimmermann, Polymers 2017, 9, 65; b) N. Bittner, H. Nefzger, G. Behnken, G. Jaeger, S. Behnken, L. Reisky, Covestro Deutschland AG, Germany . 2020, EP3587570A1.

[11] B. Krajewska, *J. Mol. Catal. B: Enz.* **2009**, *59*, 9-21.

[12] R. Greco, L. Nicolais, *Polymer* **1976**, *17*, 1049-1053.

[13] K. Nagai, K. Iida, K. Shimizu, R. Kinugasa, M. Izumi, D.-i. Kato, M. Takeo, K. Mochiji, S. Negoro, *Appl. Microbiol. Biotechnol.* **2014**, *98*, 8751-8761.

[14] J. Friedrich, P. Zalar, M. Mohorčič, U. Klun, A. Kržan, *Chemosphere* **2007**, *67*, 2089-2095.

[15] R.-J. Müller, H. Schrader, J. Profe, K. Dresler, W.-D. Deckwer, *Macromol. Rapid Commun.* **2005**, *26*, 1400-1405.

[16] a) Å. M. Ronkvist, W. Xie, W. Lu, R. A. Gross, *Macromolecules* **2009**, *42*, 5128-5138; b) H. P. Austin, M. D. Allen, B. S. Donohoe, N. A. Rorrer, F. L. Kearns, R. L. Silveira, B. C. Pollard, G. Dominick, R. Duman, K. El Omari, V. Mykhaylyk, A. Wagner, W. E. Michener, A. Amore, M. S. Skaf, M. F. Crowley, A. W. Thorne, C. W. Johnson, H. L. Woodcock, J. E. McGeehan, G. T. Beckham, *Proc. Natl. Acad. Sci. U S A* **2018**, *115*, E4350-E4357; c) S. Yoshida, K. Hiraga, T. Takehana, I. Taniguchi, H. Yamaji, Y. Maeda, K. Toyohara, K. Miyamoto, Y. Kimura, K. Oda, *Science* **2016**, *351*, 1196-1199; d) M. Furukawa, N. Kawakami, A. Tomizawa, K. Miyamoto, *Sci. Rep.* **2019**, *9*, 16038; e) R. Wei, D. Breite, C. Song, D. Gräsing, T. Ploss, P. Hille, R. Schwerdtfeger, J. Matysik, A. Schulze, W. Zimmermann, *Adv. Sci.* **2019**, *6*, 1900491; f) R. Wei, C. Song, D. Gräsing, T. Schneider, P. Bielytskyi, D. Böttcher, J. Matysik, U. T. Bornscheuer, W. Zimmermann, *Nat. Commun.* **2019**, *10*, 5581; g) V. Tournier, C. M. Topham, A. Gilles, B. David, C. Folgoas, E. Moya-Leclair, E. Kamionka, M. L. Desrousseaux, H. Texier, S. Gavalda, M. Cot, E. Guémard, M. Dalibey, J. Nomme, G. Cioci, S. Barbe, M. Chateau, I. André, S. Duquesne, A. Marty, *Nature* **2020**, *580*, 216-219; h) Y. Cui, Y. Chen, X. Liu, S. Dong, Y. e. Tian, Y. Qiao, J. Han, C. Li, X. Han, W. Liu, Q. Chen, W. Du, S. Tang, H. Xiang, H. Liu, B. Wu, *bioRxiv* **2019**, 787069.

[17] J. KubÁT, C. Pattyranie, *Nature* **1967**, *215*, 390-391.

[18] A. Vasconcelos, A. Cavaco-Paulo, *Cellulose* **2006**, *13*, 611-618.

[19] a) V. Novy, K. Aïssa, F. Nielsen, S. K. Straus, P. Ciesielski, C. G. Hunt, J. Saddler, *Proc. Natl. Acad. Sci. U S A* **2019**, *116*, 22545-22551; b) G. Vaaje-Kolstad, B. Westereng, S. J. Horn, Z. Liu, H. Zhai, M. Sørlie, V. G. H. Eijsink, *Science* **2010**, *330*, 219-222.

[20] M. Schülein, *J. Biotechnol.* **1997**, *57*, 71-81.

[21] a) S. J. Blanksby, G. B. Ellison, *Acc. Chem. Res.* **2003**, *36*, 255-263; b) Y.-R. Luo, *Handbook of Bond Dissociation Energies in Organic Compounds*, 1 ed., CRC Press, **2002**; c) I. I. Marochkin, O. V. Dorofeeva, *Comput. Theor. Chem.* **2012**, *991*, 182-191.

[22] a) R. Wolfenden, *Annu. Rev. Biochem.* **2012**, *80*, 645-667; b) T. Hentschel, H. Munstedt, *Polymer* **2001**, *42*, 3195-3203
